# Reflectance Confocal Microscopy as an Aid to Dermoscopy to Improve Diagnosis on Equivocal Lesions: Evaluation of Three Bluish Nodules

**DOI:** 10.1155/2010/168248

**Published:** 2010-09-16

**Authors:** Sara Bassoli, Stefania Seidenari, Giovanni Pellacani, Caterina Longo, Anna Maria Cesinaro

**Affiliations:** ^1^Dermatology Department, University of Modena and Reggio Emilia, Modena, Italy; ^2^Histopathology Department, University of Modena and Reggio Emilia, Modena, Italy

## Abstract

Nodular lesions can be difficult to diagnose under dermoscopy alone, since they often lack specific diagnostic features. Confocal microscopy can be used as an aid to dermoscopy, to increase the diagnostic accuracy on equivocal skin lesions. We report three cases of bluish nodular lesions, difficult to diagnose under dermoscopy alone. Confocal features were very useful in these cases to lead us to the correct diagnosis, recognizing benign versus malignant entities. Histopathology is also reported, with high correspondence compared to the confocal imaging.

## 1. Introduction

In the last decades, dermoscopy has demonstrated to be a very useful tool in the noninvasive diagnosis of skin lesions compared to clinical examination, allowing the vision of structures under the skin surface [1–4]. Dermoscopic criteria for melanocytic and nonmelanocytic lesions, as well as the ones leading to the diagnosis of benign or malignant lesions, have nowadays come straight in the daily clinical practice. Dermoscopy techniques are based on a light source, that may be polarized or non-polarized, giving rise to a coloured magnified image [5]. The digitization techniques allowed to collect lesion images to be compared and to evaluate changes over time [6, 7]. Both pattern analysis and semiquantitative algorithms [8–10] were developed, with different grades of sensitivity and specificity for diagnosis. In some cases, dermoscopy has limitations due to the paucity of dermoscopic features in certain lesions, and the differential diagnosis might be difficult, particularly in amelanotic macules and papules, or also in featureless nodules, either pigmented or non pigmented [11, 12]. 

The use of confocal microscopy in clinical practice is becoming more and more common: the commercially available tool (VivaScope 1500, Lucid Inc, Rochester, NY) is based on a laser light of 830 nm of wavelength (near-infrared), and allows the visualization of skin structures at a nearly histological resolution. A depth of 250 *μ*m can be reached, enabling the examination of the skin up to the upper dermis. Substantially, a noninvasive diagnosis is allowed, avoiding unnecessary excisions or biopsies [13, 14]. Very good correlations among the dermoscopical and confocal morphology and histopathology were demonstrated [15]. After a few years dedicated to the interpretation of confocal morphologies and the development of a glossary [16, 17], it was demonstrated [18, 19] that confocal increases the diagnostic accuracy compared to dermoscopy alone on equivocal lesions.

## 2. Materials and Methods

For the lesions described in this case series, dermoscopic images of three blue nodules were collected by the polarized dermoscope DermLite photo 3gen (San Juan Capistrano, CA), with a photocamera Canon Power Shot G10, 14,7 MegaPixels. The dermoscopic features were in all cases suspicious of malignant lesions or not clearly diagnostic; therefore, a confocal examination was performed by VivaScope 1500 (Lucid Inc., Henrietta, NY). The acquisition procedure was based on the application of a drop of water, then of an adhesive ring on the lesion; a further dermoscopic image, oriented according to the same direction of the head of the instrument, was acquired. The ring was filled with gel and the head of the instrument was positioned on it. The collection of images included three mosaics on a horizontal plane (VivaBlock modality, covering an area of 6 × 6 mm^2^), acquired at the spinous-granular layer, at the dermal-epidermal junction, in the upper dermis. Furthermore, several images of small areas (0.5 × 0.5 mm^2^) showing the most important and diagnostic features, at an increasing depth, were collected according the VivaStack modality. 

### 2.1. Case  1—A Blue Nodule on the Forearm

**Figure 1:**
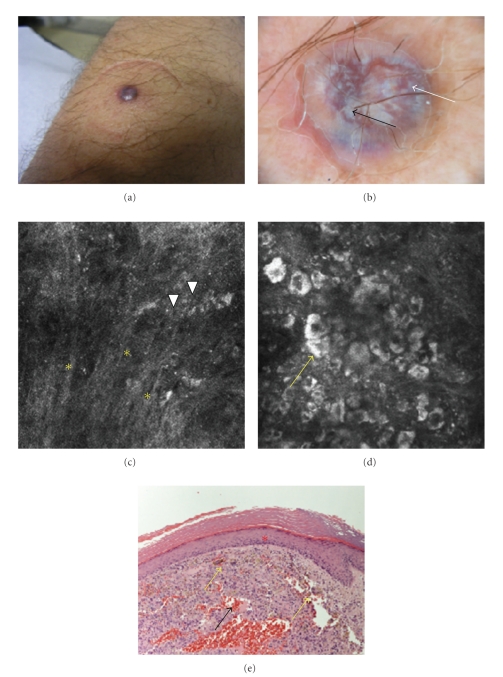
Forearm of a 54-year-old man. A hard blue asymptomatic nodule (a). Dermoscopically, the lesion showed a purple-bluish hue (white arrow), with a whitish veil and chrysalis structures (black arrow) ((b) 30x magnification). The confocal images show thick hyporeflective fibers of collagen in the dermis (yellow asterisks). Among these, hyperreflective dotted particles (white arrow heads) and several large multinucleated cells, plump and refractile, were seen (yellow arrow) ((c), (d) details 500 × 500 *μ*m). Histology revealed a fibrous hystiocytoma, with spindled fibro-hystiocytes, blood extravasation (black arrow) and numerous siderophages ((e) HH 20x magnification): in particular, plump bright multinucleated cells were identified as haemosiderophages, clearly visible in the upper portion of the dermis (yellow arrow). The flattening of the dermal-epidermal junction is highlighted by a red asterisk.

A 54-year-old man referred to our clinic for the appearance, two years before, of a bluish-purplish nodule on his right forearm. The history was of a slowly growing lesion, not painful, with a hard-elastic consistency (Figure 1(a)). Dermoscopically, the lesion was diffusely bluish-purple, with reddish nuances and a whitish veil. In the center of the lesion, some intensely white structures with well-defined borders were present, whereas at the periphery chrysalis structures were observed, corresponding to shiny, bright white, orthogonal linear streaks [20] (Figure 1(b)). The clinical and dermoscopic differential diagnosis included a hystiocytoma, a nodular melanoma and an epithelial tumor. The confocal images showed a thinned epidermis, with areas of polarization of cells along the same axis, initially suggestive of a diagnosis of pigmented basal cell carcinoma, according to the description of Nori et al. [21]. A flattening of the dermal-epidermal junction resulted in the absence of papillae (Figure 1(c)). In the superficial dermis, thick hyporeflective fibers of collagen were visible, mixed with small hyperreflective dotted particles, corresponding to leukocytic inflammatory cells (Figure 1(c)). Examining the lesion further in depth, several large multinucleated cells, plump and refractile, were seen, intermingled with smaller bright cells. These large cells were variable in shape and brightness and slightly blurred. (Figure 1(d)). The homogeneity of their content and their undefined contour, and their tendency to form plump and irregular aggregates rather than cellular nests were suggestive of the inflammatory nature of these cells. Also the typical features of a basal cell carcinoma, such as tumor islands with peripheral palisading cells, intermingled with dendritic cells and a bright inflammatory infiltrate, were absent. In spite of the suspicion of a benign lesion, the surgical excision was performed to completely clarify the diagnosis. The histologic results were a fibrous hystiocytoma, with spindled fibro-hystiocytes, blood extravasation, and numerous siderophages. Very high correspondence between confocal and histologic images was observed (Figure 1(e)); in particular, plump bright multinucleated cells were identified as haemosiderophages, clearly visible in the upper portion of the dermis. The abundant presence of collagen and of inflammatory cells associated to the blood extravasation were associated to the bluish-purple color observed in dermoscopy. 

### 2.2. Case  2—A Blue Nodule on the Back

**Figure 2:**
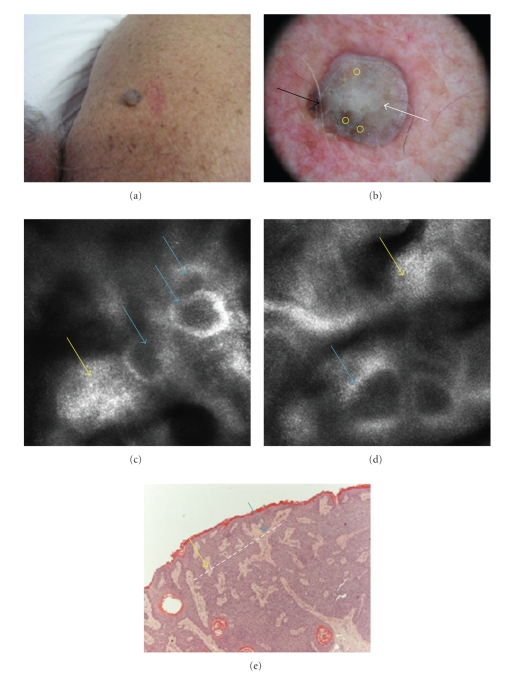
Back of a 58-year-old man. This grayish nodule of unknown history was warty and itchy (a). Dermoscopy showed a whitish veil all over the lesion (white arrow), comedo-like openings (yellow circles) and a hyperpigmented area at the periphery (black arrow) ((b) 20x magnification). The confocal examination shows the lack of the melanocytic component, bright papillary structures (blue arrows) with bulbous projections of epidermal cells, characterized by a blurred appearance (yellow arrows), due to superficial hyperkeratosis ((c), (d) details 500 × 500 *μ*m). Histology shows a seborrheic keratosis ((e) HH 10x magnification); along the horizontal section (white interrupted line), epithelial cell proliferation corresponds to bulbous projections (yellow arrow) circumscribing dermal areas corresponding to papillae (blue arrow).

A 58-year-old man was concerned about the growth of a warty lesion on his back, sometimes itchy. The lesion appeared as a grayish nodule, hard and papillomatous (Figure 2(a)). The dermoscopic imaging showed a whitish veil all over the lesion, with a hyperpigmented area at the periphery. Some comedo-like openings were also observed (Figure 2(b)). The confocal examination showed neither structures of a melanocytic lesion nor those of an epithelial tumor. Bright papillomatous structures with bulbous projections, suggestive of a seborrheic keratosis, were recognizable in spite of a blurred appearance, due to the hyperkeratotic surface of the lesion, limiting the penetration depth of the laser beam (Figures 2(c) and 2(d)). Melanocytic cells were absent. These structures corresponded to the acanthotic and papillomatous growth, typical of epithelial benign proliferations, seen in a horizontal section. The lesion was excised and histologically processed, and the pathology report was of a seborrheic keratosis (Figure 2(e)).

### 2.3. Case  3—A Bluish Nodule on the Forehead

**Figure 3:**
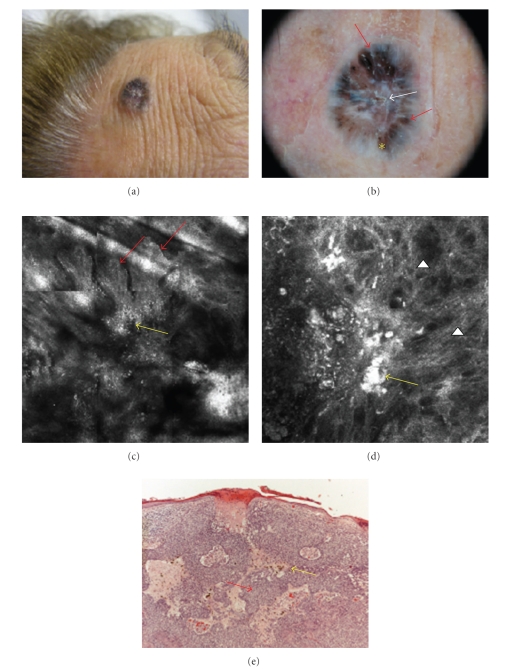
Forehead of a 75-year-old woman. A bluish, ulcerated nodule (a). The dermoscopic images were characterized by the presence of blue-brown leaf-like structures (red arrows), with an area of ulceration in the center, surrounded by white bright lines, with a chrysalis structure aspect (white arrow) and some brownish and black globular-like structures all over the lesion (yellow asterisk) (b). Confocally, reflectant tumor islands with palisading cells at the periphery ((c) red arrows) and bright cells in their inner portion ((c) mosaic 1.5 × 1.5 mm; (d) detail 500 × 500 *μ*m; yellow arrow), were present, mixed to thickened collagen fibers ((d) white arrow head). Histopathology confirmed the diagnosis of a BCC with a high correspondence, showing tumor islands ((e) HH 10x magnification, red arrow) and numerous melanophages (yellow arrow).

A 75-year-old woman referred to our observation for the growth of a bluish nodule on her forehead. The dermoscopic images were characterized by the presence of blue-brown leaf-like structures, with an area of ulceration in the center and some brownish and black globular-like structures all over the lesion. Also, white and bright chrysalis structures [20] were present under the polarized dermoscopy examination (Figure 3(a)). Confocally, reflectant tumor islands were seen, with palisading cells at the periphery and small bright cells in their inner portion, corresponding to inflammatory cells (Figures 3(b) and 3(c)). Many melanophages were also present in the upper dermis, in a context of thickened fibers of collagen (Figure 3(d)). The imaging was highly suggestive of a pigmented basal cell carcinoma, confirmed by histopathology with high correspondence (Figure 3(e)).

## 3. Discussion and Conclusion

Blue nodules are often difficult to diagnose under dermoscopy alone although it was demonstrated that this technique improves our diagnostic accuracy over naked eye examination. In histopathology, the presence of a blue color usually corresponds to a dermal inflammatory or melanocytic component, eventually associated with acanthosis and thickening in the epidermal layers [22].

The confocal examination was demonstrated to be very useful in several cases for making a differential diagnosis of nodular pigmented lesions [21, 23, 24]: basal cell carcinomas, nodular melanomas, and other entities, such as seborrheic keratosis, difficult to diagnose under dermoscopic examination alone, are usually distinguishable between each other, showing typical features. However, no evidence-based confocal criteria of benignity have been established so far for nodular lesions. Therefore, on doubtful nodular lesions, the followup has to be avoided, due to the high risk to miss a fast-growing melanoma, and the removal of lesions with suspicious clinical or dermoscopic aspects is always recommended [25, 26]. While the confocal features of basal cell carcinomas were widely described in literature, including the presence of tumor islands with peripheral palisading cells, intermingled with dendritic cells and a bright inflammatory infiltrate, the features of seborrheic keratosis were not yet systematically reviewed although some cases were reported in the differential diagnosis with other pigmented and nonpigmented entities [27].

Since in the three bluish nodules we collected, dermoscopy did not clarify completely the diagnostic doubts, a confocal examination was performed. The lack of atypical cells in the superficial layers and of a melanocytic component was diriment for the diagnosis of benign entities in lesions 1 and 2. In particular, the dermal component constituted of large plump bright cells in the hystiocytoma of the forearm was highly correspondent to the histopathology report. Due to the limited penetration depth of the instrument, the examination of the dermatofibromas is not always discriminant for the diagnosis, since these are mainly dermal lesions. Confocal studies on dermatofibromas are therefore still missing.

In the seborrheic keratosis of the back, the bluish color was related to the presence of epidermal thickening and acanthosis, and the confocal showed a regular architecture with enlarged bulbous projections of the epidermis. These features had high correspondence with histopathology. The confocal features of basal cell carcinoma, including tumor islands with palisading cells and dendritic cells, and inflammatory infiltrate were relevant for diagnosis in the case of the bluish nodule on the forehead. 

In agreement with the most recent studies [18, 19], we can affirm that, together with the clinical and dermoscopical examination, the confocal microscopy is a relevant aid in the daily practice of a dermatologist. The acquisition and the interpretation of confocal images require a long training for the operators but allow a nearly histological visualization of equivocal lesions and a very accurate pre-operative diagnosis.
